# Determinants of asthma among adults in Tigray, Northern Ethiopia: a facility-based case-control study

**DOI:** 10.7717/peerj.16530

**Published:** 2024-01-05

**Authors:** Tirhas G. Gebresillasie, Alemayehu Worku, Ahmed Ali Ahmed, Negussie Deyessa Kabeta

**Affiliations:** 1 Department of Public Health, College of Health Sciences, Aksum University, Axum, Tigray, Ethiopa; 2 School of Public Health, College of Medicine and Health Science, Addis Ababa University, Addis Ababa, Ethiopia

**Keywords:** Adult, Case-control, Tigray, Ethiopia, Determinants, Behavioral, Asthma, Allergic, Environmental

## Abstract

**Background:**

Asthma is a public health concern affecting millions of productive age groups. Several studies were conducted on the determinants of asthma in children. However, little is known about the determinants of asthma among adults in Ethiopia. Understanding the determinants of asthma among adults can help reduce its burden. This study was aimed at identifying determinant factors for developing asthma among adults in Tigray hospitals.

**Methods:**

A facility-based, unmatched case-control study design was conducted from January 1 to April 26, 2019. A total of 698 participants (228 cases and 470 controls) completed their guided interviews using structured and pretested questionnaires by trained data collectors. A modified standard questionnaire from the European Community Respiratory Health Survey II (ECRHS II) was used to collect the data. The case definition was patients having asthma, and the control definition was patients without asthma. Data were entered and cleaned using Epi Data Manager Version 3.1 software and imported to statistical packages for social sciences Version 25 software for analysis. To identify asthma determinants, bivariate and multivariable logistic regression models were fitted.

**Results:**

The response rate for both cases and controls was 95.9%. The odds of developing asthma was nearly twice higher among those who resided in urban (AOR = 1.68; 95% CI [1.13–2.50]), more than twice higher among those who have income less than 1000 ETB (AOR = 2.3; 95% CI [1.17–4.56]), twice higher among those who had history of skin allergy (AOR = 2.09; 95% CI [1.14–3.86]), over four times higher among those with family history of asthma (AOR = 4.26; 95% CI [2.63–6.91]), three times higher among those having house dust or smoke exposure (AOR = 3.01; 95% CI [1.96–4.64]), over five times higher among those lifetime firewood users (AOR = 5.39; 95% CI [3.34–8.72]), door opening while cooking (AOR = 0.35; 95% CI [0.26–0.55]), nearly two times higher among those having house dampness (AOR = 1.98; 95% CI [1.069–3.68]), over seven times higher among pet owners (AOR = 7.46; 95% CI [4.04–13] and almost twice higher among those who were physically inactive (AOR = 1.75; 95% CI [1.11–2.85]).

**Conclusion:**

Asthma has been associated with urbanization, low income, a history of allergic diseases, indoor smoke or dust, firewood use, pet ownership, and a sedentary lifestyle. The community should be informed about the known risks and implement preventive steps like opening a door while cooking to lower the risk of asthma.

## Introduction

Asthma is a condition in which airways get narrow and swell and produce extra mucus that can make breathing difficult and trigger coughing, wheezing, and shortness of breath. Asthma is a public health concern affecting millions of productive age groups in developed and developing countries with prevalence rates 1 to 21% among adults (Asher & Pearce, 2014; Enilari & Sinha, 2019).

Asthma affects roughly 334 million people worldwide, and if the current trend continues, there will be a 25% increase in asthma cases over the next fifteen years, leading to numerous annual deaths. Asthma causes less than 1% of deaths, which is a low mortality rate compared to other chronic diseases but it causes 250,000 potentially avoidable fatalities worldwide (Ellwood et al., 2017).

Asthma was first identified as a disease in hospital OPD visits and admissions in Ethiopia after the middle of the 1970s, according to the EPHA and FMOH 2014-2016 National Strategic Action Plan (NSAP) (Ethiopian Public Health Association, 2012; Federal Ministry of Health Ethiopia, 2016). Despite limited documented research on the determinants of asthma, prevalence studies found varying prevalence rates. A systematic review of major NCDs epidemiology (Misganaw et al., 2014) as well as recent prevalence, and associated factor studies in Debrebirhan and Jimma town reported 29.6% and 4.9% rates, respectively (Tefereedgn & Ayana, 2018; Shine, Muhamud & Demelash, 2019).

Both in developed and developing nations, asthma causes considerable economic and social costs. The majority of the burden of asthma manifests as disability in those under 45 years old, but in older people, inadequate long-term care and a delay in receiving assistance during an attack are linked to premature mortality. As a result, asthma has significant negative economic, medical, and social effects on the patient, their family, and society, evidenced by job and school absenteeism, frequent ER visits, hospitalizations, and even mortality at any age (Cruz et al., 2010; Enilari & Sinha, 2019; Alem, Gebeyehu & Arega, 2020).

In order to achieve optimal asthma control, GINA and other stakeholders developed and routinely reviewed fixed international scientific treatment recommendations. Asthma was also listed as a priority in Ethiopia’s National Strategic Action Plan for preventing and controlling NCDs (Federal Ministry of Health Ethiopia, 2016). The document on the clinical and programmatic management of significant NCDs was also created in Ethiopia in 2016 based on the progress of the achievement of the global 2025 NCDs objective (Federal Ministry of Health Ethiopia, 2016). Asthma is still a public health concern in many regions of the world despite the local and international efforts done to reduce the disease’s burden.

Designing, organizing, and putting into practice prevention efforts to lessen the burden of asthma can be based on an evidence-based understanding of the modifiable determinants of the disease development. To the best of our knowledge, there have not been enough studies on the determinants of developing asthma in Ethiopia, particularly in the Tigray region. Therefore, this study was carried out to identify determinants of asthma among adult patients attending in the OPD of Tigray hospitals. This study can be used as a baseline for interested researchers, policymakers, planners, and implementers working on asthma prevention and control programs.

## Material and Methods

### Study area and setting

A facility-based unmatched case-control study was conducted in four hospitals namely “Suhul, St Merry, Axum Referral, and Adwa hospitals”, located in Tigray regional state, Northern Ethiopia. The region is one of the nine regional states of Ethiopia. It is divided into seven zones with 52 Woredas and each zone is homogenous in terms of socio-economic, access, and availability of health services. The region has climatic characteristics of kola (semi-arid) 39%, Woinadega (warm temperate) 49%, and Dega (temperate) 12% (Tigray Regional Health Bureau, 2016). The total population of the region is 5,443,000 (49.2% male and 50.8% female) according to the 2019 population projection (UNICEF, 2019).

All adult patients (with and without asthma) who visited the selected governmental hospitals during the data collection period were the source of the population. Adult patients aged 18 years or older diagnosed with asthma were considered as cases and adult patients without asthma but visiting the selected hospital with other diseases during the study period were considered as control. Both cases and controls were selected using their medical record during their visit to the hospitals.

Confirmed cases of bronchiectasis, chronic bronchitis, chronic obstructive pulmonary disease, emphysema, lung cancer, cardiac illness, and diabetes were excluded from the study because these conditions have shared risk factors. Patients with significant respiratory distress due to an exacerbation requiring an ED visit or admission were excluded from the study and were guided to see a physician or assigned health professional to get help for their problems. For each study participant, the interview was conducted while waiting for the hospital’s regular service or exit.

### Sample size and sampling techniques

The sample size was determined with the assumption of 80% power, 5% level of significance, control-to-case ratio 1:2, and 10% non-response rate taken from previous similar studies (Ibrahim, 2013; Verma & Pradesh, 2014; Fu et al., 2016). After calculating the sample size for each essential factor, the maximum calculated sample size was considered the final sample size. Thus, the final sample size was 728 (243 cases and 485 controls). Each study participant was selected through a systematic sampling technique with an interval of every third adult patient who visited the adult OPD in the selected hospitals (Table S1).

### Data collection procedure

Data were collected through face-to-face interviews using structured and pretested questionnaires. Patient’s medical records were used to select the cases and controls. Both the cases and the controls responded to the questionnaire. The questionnaire was adopted from ECRHS II (European Community Respiratory Health Survey II Steering Committee, 2002) containing four major sections. The first section of the questionnaire was related to socio-demographic and allergy-disease-related factors. Environment-related questions were covered in the second and third sections of the questionnaire. The fourth section included questions that were behavior-related. The second, third, and fourth sections are the potential risk factor predictors. Serious asthmatic patients who required an ER visit or admission were excluded from the study and were advised to contact a doctor for symptom treatment.

### Data processing and statistical analysis

The collected data were checked, coded, and entered into Epi-data Manager version 3.1 and then exported to SPSS version 25 for analysis (SPP Inc., Chicago, IL, USA). The entered data were cleaned and edited before subsequent analysis. Cross tabulations and summary statistics like mean and standard deviation were carried out. Bivariate and multivariable logistic regression models were fitted to identify the relationship between the independent variables (socio-demographic, allergy-disease related, environmental, and behavioral factors) and the dependent variable (asthma presence). Sex, residence, marital status, education, occupation, income, history of nasal allergy, skin allergy, family history of asthma, family history of skin/nasal allergy, exposure to freezing weather, NSAIDs use, seeking medical help, and history of severe respiratory infection in early childhood were among the first section of the questionnaire analyzed in the bivariate model. Job status, dust or smoke exposure in the house, outdoor dust or smoke exposure, proximity to road vehicle traffic, lifetime firewood use for cooking, presence of any dampness inside the house, door opening while cooking, having a separate room for cooking, possessing of pets, exposure to animal dander, presence of pets during childhood, physical inactivity, packed food use, BMI, vegetable and fruit use, alcohol use, ever smoked for as long as a year, presence of regular second smoke and non-smoke tobacco use were from environmental and behavioral variables fitted to the bivariate model. All variables with *P* < 0.2 in the bivariate logistic regression model were included in the multivariable logistic regression. Variables associated with asthma were determined in multiple logistic regression at *P*-value <0.05 and AOR of 95% CI. The model fitness was checked using the Hosmer and Lemeshow goodness of fit test statistic (*P* = 0.698).

Ethical clearance was obtained from the Research Ethical Committee (REC) of the School of Public Health and the IRB (Institutional Review Board) of the College of Health Sciences at Addis Ababa University with approval number 004/18/SPH. Moreover, official permission was secured from the Tigray regional health bureau. Written informed consent from participants was also gained before conducting the study. Participants’ information obtained from the questionnaire was kept confidential using data coding. Participants were also informed that participation was on voluntary basis.

### Operational definition

#### Asthma:

is defined based on the diagnosis of a clinician in the health facility.

#### Door opening while cooking:

ask respondents if they open their door or window while cooking.

#### Lifetime firewood/coal users:

if respondents use wood, cow dung, or charcoal as a source of energy for cooking either formerly or currently or both.

#### House dampness or mold:

asking respondents if there is water damage, dampness, visible mold and mold odor in their house.

#### Physical inactivity:

Based on the World Health Organization definition failure to perform at least 150–300 min of moderate-intensity aerobic physical activity; or at least 75–150 min of vigorous-intensity aerobic physical activity; or an equivalent combination of moderate- and vigorous-intensity activity throughout the week.

#### Possession of pet:

if respondents keep pets in their house such as cats, dogs.

## Results

### Descriptive analysis

#### Socio-demographic and asthma related characteristics of respondents

Out of the entire 728 scheduled sample members, 698 (228 cases and 470 controls) participated in the interview, yielding a 95.9% response rate. More than half of the respondents, 60.7% of both cases and controls were males. The age of study subjects ranged from 18 to 89 (mean 41, SD 15.07) years, in which 46.7% of the cases and almost half of the respondents of the controls were in the age group of 35–55 years. The majority, 85% of cases and 86.8% of controls were orthodox in religion, and almost 98% were Tigrian by ethnicity. About 64% of cases and 58.3% of the controls were married; 32% of cases and 36% of controls were illiterate whereas the majority, 64% of cases and 51% of controls, are urban dwellers. More than half 53.5% of the cases and 66% of controls had medium household income (Table 1).

**Table 1:** Socio-demographic and asthma-related characteristics of respondents by asthma status in Tigray Hospitals, Northern Ethiopia, 2019 (*n* = 698).

**Characteristics (*n* = 698)**	**Category**	**Asthma status**		**Total *n* (%)**
		**Cases *n* (%)**	**Controls *n* (%)**	
Sex	Female	94 (41.2)	180 (38.3)	274 (39.3)
	Male	134 (58.8)	290 (61.7)	424 (60.7)
Age	18–34	75 (32.9)	193 (41.1)	268 (38.4)
	35–55	123 (53.9)	203 (43.2)	326 (46.7)
	Above 55	30 (13.2)	74 (15.7)	104 (14.9)
Religion	Orthodox	195 (85.6)	402 (85.5)	597 (85.5)
	Muslim	33 (14.4)	68 (14.5)	101 (14.5)
Ethnicity	Tigrian	219 (96)	454 (96.5)	684 (98.0)
	Other	9 (4)	16 (2.3)	14 (2.0)
Marital status	Married	146 (64)	274 (58.3)	420 (60.2)
	Unmarried	82 (36)	196 (41.7)	278 (39.8)
Education	Illiterate	72 (31.6)	171 (36.4)	243 (34.8)
	Primary school	56 (24.6)	111 (23.6)	167 (23.9)
	Secondary school and above	100 (43.9)	188 (40)	288 (41.3)
Residence	Urban	146 (64)	240 (51.1)	386 (55.3)
	Rural	82 (36)	230 (48.9)	312 (44.7)
Monthly Income	<1,000	36 (15.8)	30 (6.4)	66 (9.5)
	1,000–4,000	122 (53.5)	308 (65.5)	430 (61.6)
	>4,000	70 (30.7)	132 (28.1)	202 (28.9)
Occupation	Unemployed	65 (28.5)	165 (35.2)	230 (33)
	Employed	163 (71.5)	305 (64.8)	85 (12.2)
History of Asthma in years (*n* = 228)	1–15 years	33 (14.5)	NA	33 (14.5)
	16–30 years	85 (37.3%)	NA	85 (37.3)
	More than 30 years	110 (48.25)	NA	110 (48.2)
Asthma attack last 12 months (*n* = 228)	Yes	212 (92.9)	NA	210 (96.3)
	No	16 (7.01)	NA	18 (3.7)
Asthma control level (*n* = 228)	Well-controlled	77 (33.7)	NA	77 (33.7)
	Less controlled	102 (44.7)	NA	102 (44.7)
	Uncontrolled	49 (21.5)	NA	49 (21.5)
Asthma during the menstrual cycle (*n* = 107)	Gets Better	0	NA	0
	Gets Worse	6 (5.6)	NA	6 (5.6)
	No change	101 (94.4)	NA	101 (94.4)
Asthma during pregnancy (*n* = 107)	Gets Better	3 (2.8)	NA	3 (2.8)
	Gets Worse	29 (27)	NA	29 (27)
	No change	75 (70.2)	NA	75 (70.2)

**Notes.**

NA Not applicable

Out of all study participants, it was shown that 58.8% of males and 41.2% of females had asthma. Of the 228 asthma cases, 48% had the condition for more than 30 years. The majority of cases (92.9%) had experienced at least one-time asthma symptom in the last 12 months. Regarding self-reported asthma control level, only 33.3% of the cases had a good control level of asthma in the previous three months. Looking at female asthmatic subjects, only 5.6% got their asthma worse with their monthly menstrual cycle, and 27% of females got their asthma worse during their pregnancy (Table 1).

#### Environmental and behavioral characteristics of the study participants

Small proportion: 26% reported that they experienced job-related respiratory problems and a significant proportion of the participants, 64% of them, used lifetime firewood/coal as a source of energy for cooking. Looking at carpet use, 80% of the participants do not have carpets in the mostly used room, and 11.3% of the carpets used stayed from 1 to 5 years. A total of 11.3% of the study participants possess pets, and from them, 93.6% of the pets are allowed to stay in the participant’s bedroom; 39.5% of the study participants own domestic animals such as cows, sheep, and goats, and 92.3% of the owners keep their domestic animals in a particular place. Concerning the behavioural factors, 26.4% drink alcohol, and only 4.2% of the study participants have a history of cigarette smoking (Table 2).

### Predictors of asthma

Sex, age, residence, household income, ever had skin allergy, exposure to freezing weather, NSAIDs use, family history of asthma, family history of skin allergy, seeking medical help, history of severe respiratory infection in early childhood, dust/smoke inside the house, dust/smoke outside the house, the proximity of road traffic, door opening while cooking, lifetime firewood/coal use, having a separate cooking room, possession of pets, physical inactivity and packed food use were statistically associated with asthma in the bivariate logistic regression analysis model.

Variables which were significantly associated with asthma in the bivariate model were entered into multiple logistic regression analyses to control possible confounding factors. Urban residence, income less than 1,000 ETB, history of skin allergy, family history of asthma, house dust or smoke exposure, lifetime firewood use, door opening, house dampness, owning pets, and being physically inactive were found to be significant at *p* value <0.05 and 95% CI.

**Table 2:** Environmental and behavioural characteristics of respondents by asthma status in Tigray Hospitals, Northern Ethiopia, 2019 (*n* = 698).

**Characteristics**	**Category**	**Asthma status**		**Total (%)**
		**Cases *n* (%)**	**Controls *n* (%)**	
Ever had any job-related respiratory problem	Yes	99 (43.4)	84 (17.9)	26.2
	No	129 (56.6)	386 (82.1)	73.8
Firewood use for cooking	Yes	152 (66.7)	300 (63.8)	64.8
	No	76 (33.3)	176 (36.2)	35.2
Carpet use in the living room	Yes	47 (20.6)	92 (19.6)	19.9
	No	181 (79.4)	378 (80.4)	80.1
Carpet year of the Living Room	less than 1 year	13 (5.7)	31 (6.6)	6.3
	1–5 years	26 (11.4)	53 (11.3)	11.3
	Above 5 years	9 (3.9)	7 (1.5)	2.3
	No Carpet	108 (78.9)	379 (80.6)	80.1
Possession of pets	Yes	56 (24.6)	23 (4.9)	11.3
	No	172 (75.4)	447 (95.1)	88.7
Pets allowed in the bedroom	Yes	53 (67)	21 (26.6)	93.6
	No	3 (3.8)	2 (2.5)	33.3
Presence of Domestic animals	Yes	92 (40.4)	184 (39.1)	39.5
	No	136 (59.6)	286 (60.9)	60.5
Separate place for domestic animals	Yes	85 (92.4)	159 (86.4)	92.3
	No	7 (5.5)	25 (13.7)	7.7
Alcohol use	Yes	65 (28.5)	119 (25.3)	26.4
	No	163 (71.5)	351 (74.7)	73.6
Ever smoked cigarettes	Yes	13 (5.7)	16 (3.4)	4.2
	No	215 (94.3)	456 (96.6)	95.8
Second smoke exposure	Yes	18 (7.9)	23 (4.9)	5.9
	No	210 (92.1)	447 (95.1)	94.1
Childhood pet ownership	Yes	107 (46.9)	197 (41.9)	43.6
	No	121 (53.1)	273 (58.1)	56.4
Vegetable or fruit use	Yes	67 (29.4)	156 (33.2)	31.9
	No	161 (70.6)	314 (66.8)	68.1
Snack use	Yes	64 (29.2)	155 (70.8)	32.7
	No	158 (33.2)	315 (65.8)	57.3

The multiple logistic regression analyses showed that the odds of getting asthma among urban residents were 1.68 times higher than rural residents (AOR = 1.68; 95% CI [1.13–2.50]), the odds of getting asthma among respondents with monthly household income less than 1000ETB is 2.3 times higher than respondents with higher household monthly income (AOR = 2.3; 95% CI [1.17–4.56]). Moreover, the odds of getting asthma among respondents who had a history of skin allergy is 2 times higher compared to their peers (AOR = 2.09; 95% CI [1.14–3.86]), the odds of getting asthma among respondents who had a family history of asthma is 4.3 times higher compared to their peers (AOR = 4.26; 95% CI [2.63–6.91]). The odds of getting asthma among respondents exposed to dust/smoke in the house were three times higher than respondents who were not exposed to dust/ smoke in the house (AOR = 3.01; 95% CI [1.96–4.64]). Similarly, the odds of getting asthma among respondents who used firewood/coal in their lifetime were 5.4 times higher than those who do not use (AOR = 5.39; 95% CI [3.34–8.72]); however the odds of getting asthma among respondents who open their door were 35 percent lower than respondents who were not opening their door while cooking which is a protective factor (AOR = 0.35; 95% CI [0.26–0.55]). In regard to dampness, the model identified that the odds of getting asthma among respondents who have dampness inside their houses were 1.9 times higher than respondents who were no having dampness inside their houses (AOR = 1.98; 95% CI [1.07–3.68]). Cognizant with this, the odds of getting asthma among respondents who possess pets were 7.5 times higher than respondents who do not possess pets (AOR = 7.48; 95% CI [4.04–13.82]). The odds of getting asthma among physically inactive respondents were almost 1.8 times higher compared to their peers (AOR = 1.75; 95% CI [1.08–2.85]) (Table 3).

**Table 3:** Multivariable logistic regression analysis of socio-demographic, allergic, environmental, and behavioral determinants of asthma among adults in Tigray Hospitals, Northern Ethiopia, 2019 (*n* = 698).

**Variables**	**Category**	**Asthma status**		**AOR**	***P* value**
		**Cases (%)**	**Controls (%)**		
Residence	Urban	146 (64)	240 (51.1)	1.68 (1.13–2.5)	0.011
	Rural	82 (36)	230 (48.9)	1	
Income (ETB)	<1,000	36 (15.8)	30 (6.4)	2.3 (1.17–4.56)	0.016
	1,000–4,000	122 (53.5)	308 (65.5)	0.75 (0.52–1.25)	0.33
	>4,000	70 (30.7)	132 (28.1)	1	
Ever had a skin allergy	Yes	36 (15.8)	36 (7.7)	2.09 (1.14–3.86)	0.018
	No	192 (84.2)	434 (92.3)	1	
Family history of asthma	Yes	81 (63.8)	46 (36.2)	4.26 (2.63–6.91)	0.000
	No	147 (25.7)	424 (74.3)	1	
Dust in the house	Yes	34 (14.9)	81 (46)	3.01 (1.96–4.64)	0.000
	No	194 (85.1)	389 (74.5)	1	
Firewood/coal use	Yes	177 (77.6)	270 (57.4)	5.39 (3.34–8.72)	0.000
	No	51 (22.4)	200 (42.6)	1	
Dampness in the house	Yes	34 (17.5)	38 (8.1)	1.98 (1.07–3.68)	0.030
	No	194 (82.5)	432 (91.9)	1	
Road traffic proximity from your house in minute	<2 Min	62 (27.2)	93 (19.8)	1.49 (0.94–2.35)	0.088
	>2 min	166 (72.8)	377 (80.2)	1	
Door opening while cooking	Yes	92 (40.4)	104 (22.1)	0.35 (0.23–0.55)	0.000
	No	136 (59.6)	366 (77.9)	1	
Pet possession	Yes	56 (24.6)	23 (4.9)	7.48 (4.04–13.82)	0.000
	No	172 (75.4)	447 (95.1)	1	
Inadequate physical activity	Yes	57 (25)	75 (16)	1.75 (1.08–2.85)	0.002
	No	171 (75)	395 (84)	1	

## Discussion

The current study identified different factors that are probably associated with the onset of asthma in the study area. Urban residence, low monthly income, history of skin allergy, family history of asthma, house dust or smoke exposure, lifetime firewood use, house dampness, owning pets and being physically inactive were significant predictors for the development of asthma. On the other hand door opening while cooking was a protective factor for asthma.

Urban residence was positively associated with asthma. The relationship could be urban dwellers might get exposed to air pollution from automobiles, industrial emissions, and solid and liquid wastes that can trigger bronchial irritation. As a result, the bronchial irritation caused due to outdoor air pollution may mimic asthma in susceptible individuals. Asthma may have been caused as a result of this condition. This study agrees with the studies done in Canada, India, Uganda, and Ethiopia (Aggarwal et al., 2006; Santos et al., 2018; Kirenga et al., 2019; Shine, Muhamud & Demelash, 2019). On the other hand, the present study result was inconsistent with other studies done in Canada and India, where the risk of having asthma was significantly higher in respondents who were residing in rural areas than their counterparts (Gupta & Mangal, 2006; Crighton, Wilson & Senécal, 2010). This variation might be due to the difference in the study subjects’ lifestyle where the study in Jaipur district India suggested that the higher prevalence of asthma was observed in rural areas related to hookah smoking.

In the current study, the odds of getting asthma among respondents with a monthly income less than 1,000 ETB were more than twice higher than their peers. The reason for this is that people with low monthly wages have fewer alternatives for implementing asthma prevention techniques (Agrawal, Pearce & Ebrahim, 2013). This result agrees with studies conducted in Korea, Sweden, Australia, New York, and Ethiopia (Claudio, Stingone & Godbold, 2006; Hedlund, Eriksson & Rönmark, 2006; Kozyrskyj et al., 2010; Choi et al., 2012).

This study revealed that the odds of getting asthma among respondents who have a history of skin allergy were higher as compared to their counterparts. The association could be several immunological and structural skin cells, including T cells, are stimulated by allergens due to the manifestation of skin allergy. These cells release mediators for the stimulation of cell-mediated responses and the production of allergen-specific antibodies. In response to the particular allergen that may causes asthma, the presence of a skin allergy could trigger bronchial inflammation. Similar associations have been also observed in several studies (Aggarwal et al., 2006; Gupta & Mangal, 2006; Rémen et al., 2012; Kirenga et al., 2019).

The current finding showed that having a family history of asthma increases the odds of getting asthma. There are two probable explanations for this association: either inherited factors or a shared environment amongst family members contribute to the pathophysiology of asthma. This implies that a susceptible individual with a family history of asthma has to minimize the environmental conditions that can trigger the development of asthma signs and symptoms. This result agreed with the study done in Colombia, India, Uganda, and Ethiopia (Aggarwal et al., 2006; Liu et al., 2009; Gonzalez-Garcia et al., 2015; Wortong, Chaiear & Boonsawat, 2015; Shine, Muhamud & Demelash, 2019; Abebe et al., 2021).

In this study, door opening while cooking was found to be a protective factor for asthma. Not opening the door could result in poor ventilation of the house, which could cause respiratory tract congestion due to the possibility of regular usage of firewood/coal. This result suggests that to promote a healthy indoor air exchange while cooking requires door opening. This result is consistent with a study done in Pakistan and Ethiopia (Khan et al., 2014; Abebe et al., 2021). On the other hand, this study is inconsistent with the study done in the Netherlands which mentioned that there was no relationship between kitchen ventilation and asthma (Willers et al., 2006). This discrepancy may result from differences in the frequency and length of door openings during cooking, methodology, and study population. For instance, the study from the Netherlands recruited five-year-old children who had no connection with cooking.

In the present study, an association was observed among respondents who used lifetime firewood/coal as a source of energy for cooking. The connection between the two can be explained by the likelihood that burning wood inside the house can cause irritation to the bronchi that might mimic the onset of asthma. This is because various inflammatory substances are released, such as cytokines, and chemokines, which can cause inflammatory damage and increase bronchial reactivity. It can even get worse cooking food with firewood/coal without opening a door due to indoor air pollution. Repeated exposure to coal or wood smoke irritates the lungs and increases the risk of developing asthma. This result is similar to Nigeria, Peru, USA and Ethiopia (Thacher et al., 2013; Gaviola et al., 2016; Enilari & Sinha, 2019; Abebe et al., 2021) but inconsistent with the studies done in Colombia, China, India, and Uganda (Barry et al., 2010; Guddattu, Swathi & Nair, 2010; Jie et al., 2016; Kirenga et al., 2019). This variation could be due to difference in the sample size, cooking space, door opening frequency, and duration of exposure to firewood/coal.

The odds of getting asthma among respondents who have dampness or molds inside their houses were higher compared to those who do not have dampness or molds inside their houses. The association between mold and asthma can be explained by the fact that indoor molds can trigger allergic reactions mediated by immunoglobulin E, toxic reactions brought on by non-specific inflammatory reactions and irritating volatile organic compounds produced by microbes or cell walls (Hwang, Liu & Huang, 2011). The association can be also explained if molds and humidity are inside the house which may produce unpleasant odors that irritate the respiratory system and may precipitate the onset of asthma in a susceptible individual. This finding is similar to the study done in Taiwan, USA, Singapore and Netherlands (Tham et al., 2007; Hwang, Liu & Huang, 2011; Weinmayr et al., 2013; Xiao et al., 2021).

The odds of getting asthma among respondents who possessed pets were higher compared to those who do not possess pets. This may be because the respondents may not know the risks of pet keeping for asthma which was mentioned in research done in China and Bulgaria where the percentage of people who had avoidance behavior towards pets was low. The low avoidance behavior of pets was similar in this study where 93.6% of the pets were allowed to stay in the respondent’s bedroom. This finding was similar to the study done in Finland, China, and Congo (Jaakkola et al., 2002; Takkouche et al., 2008; Jie et al., 2016; Obel et al., 2017; Luo et al., 2018). On the other hand, this finding was inconsistent with the study done in Japan which reported that having pets was a protective factor (Taniguchi & Kobayashi, 2023). This variation could be due to difference in study population, pet ownership definition and duration of pet ownership which was not considered in the current study.

The odds of getting asthma among physically inactive respondents were higher compared to those who were physically active. This finding was consistent with a study done in America and Sweden (Eijkemans et al., 2012; Agrawal, Pearce & Ebrahim, 2013; Jerning et al., 2013; Garcia-Aymerich et al., 2014; Ebell, Marchello & O’connor, 2017) but inconsistent with the study done in Spain (Benet et al., 2011; Ebell, Marchello & O’connor, 2017). This variation might be due to the difference in study populations and measurement tools used for physical inactivity.

## Conclusion

The study found various modifiable determinants of adult asthma in the Tigray region. Keeping the door open while cooking was found to be a protective factor. Asthma has been associated with urbanization, low income, history of allergic disorders, exposure to house dust or smoke, use of firewood, ownership of pets and sedentary lifestyle. Health professionals should screen and inform patients who are at higher risk to asthma. Moreover, community and personal sensitization through information, education, and communication strategies about the identified determinant factors and implement preventive steps like opening a door while cooking, being physical active and minimizing firewood use might help to lower the risk of asthma among adults. Policymakers, researchers and clinicians involved in the field will be interested in a larger prospective studies.

## Limitation

This study shares the weakness of the case-control study which is recall bias that may exaggerate or reduce the association. Moreover, respondents were asked about both disease outcomes and risk factors at a time. So it is difficult to conclude the direction of causality in this study.

##  Supplemental Information

10.7717/peerj.16530/supp-1 Table S1 Sample size calculation for Different scenarios and risk factors

10.7717/peerj.16530/supp-2 Table S2 Bivariate logistic regression analysis of socio demographic and allergic variables

10.7717/peerj.16530/supp-3 Table S3 Bivariate logistic regression analysis of Environmental and Behavioral variables

10.7717/peerj.16530/supp-4 Dataset S1 SPSS raw data

## Additional Information and Declarations

### Data Availability

The following information was supplied regarding data availability: The raw data are available in the Supplemental File.
