# Supplementary material for: Determinants of asthma among adults in Tigray, Northern Ethiopia: a facility-based case-control study
Source: PeerJ. 2024 Jan 5;12:e16530. doi: 10.7717/peerj.16530 (PMC10773448; doi:10.7717/peerj.16530)
Supplement: Table S1 [file peerj-12-16530-s001.docx]

**Table 1: Sample size calculation for Different scenarios and risk factors**

| **Factor** | **% of controls exposed** | **% of cases exposed** | **OR** | **Calculated the number of cases** | **Calculated the number of controls** | **Total sample size (with 10% NRR)** | **References used** |
| --- | --- | --- | --- | --- | --- | --- | --- |
| **Smoking** | 16.6 | 26.3 | 1.8 | 243 | 485 | 728*** | (Qing Ling Fu, 2016) |
| **Occupation** | 35.8 | 54.7 | 2.2 | 88 | 176 | 291 | Ibrahim, 2013 & Anil et al 2011 |
| **Education** | 62 | 83 | 2.99 | 61 | 122 | 201 |  |
| **Parental atopy** | 25.5 | 54.7 | 3.5 | 37 | 73 | 121 |  |
| **Family history of asthma** | 17 | 50.9 | 5.0 | 26 | 51 | 85 |  |

*** The actual sample size for this study
