# Supplementary material for: Determinants of asthma among adults in Tigray, Northern Ethiopia: a facility-based case-control study
Source: PeerJ. 2024 Jan 5;12:e16530. doi: 10.7717/peerj.16530 (PMC10773448; doi:10.7717/peerj.16530)
Supplement: Table S2 [file peerj-12-16530-s002.docx]

**Table 2: Bivariate Logistic regression analysis of Socio-demographic and Allergy- disease-related variables assessed as determinants of Asthma among Adults in Tigray Hospitals, Northern Ethiopia, 2019 (n=698)**

| **Variables** | **Category** | **Asthma Status** | | **COR [95 % CI]** | **P value** |
| --- | --- | --- | --- | --- | --- |
|  |  | **Cases (%)** | **Controls (%)** |  |  |
| **Residence** | Urban | 146 (64) | 240 (51.1) | 1.71 (1.23-2.36) | 0.001 |
|  | Rural | 82(36) | 230(48.9) | 1 |  |
| **Age** | 18-34 | 75(32.9) | 193 (41.1) | 1 |  |
|  | 35-55 | 123(53.9) | 203 (43.2) | 1.56 (1.101-2.21) | 0.012 |
|  | Above 55 | 30(13.2) | 74 (15.7) | 1.043 (0.63-1.72) | 0.87 |
| **Income (ETB)** | <1000 | 36(15.8) | 30(6.4) | 2.26 ( 1.29-3.98) | 0.005 |
|  | 1000-4000 | 122(53.5) | 308(65.5) | 0.75 ( 0.52-1.07) | 0.11 |
|  | >4000 | 70 (30.7) | 132(28.1) | 1 |  |
| **Ever had a nasal allergy** | Yes | 73(32) | 108(23) | 1.58 (1.11-2.24) | 0.01 |
|  | No | 155(68) | 362(77) | 1 |  |
| **Ever had skin**  **Allergy** | Yes | 36(15.8) | 36(7.7) | 2.26 (1.38-3.69) | 0.001 |
|  | No | 192(84.2) | 434(92.3) | 1 |  |
| **Family history of asthma** | Yes | 81 (63.8) | 46 (36.2) | 5.08(3.38-7.64) | 0.000 |
|  | No | 147 (25.7) | 424 (74.3) | 1 |  |
| **Family history of skin allergy** | Yes | 50 (21.9) | 39 (8.3) | 3.10 (1.97-4.89) | 0.000 |
|  | No | 178 (78.1) | 431 (91.7) | 1 |  |
| **History of exposure to freezing weather** | Yes | 28 (12.3) | 22(4.7) | 2.85 (1.59-5.11) | 0.000 |
|  | No | 200 (87.7) | 448 (95.3) | 1 |  |
| **Seeking medical help for respiratory problems** | Yes | 82 (36) | 129(27.4) | 0.67 (0.48-0.94) | 0.022 |
|  | No | 146(64) | 341(72.6) | 1 |  |
| **Non-steroidal anti-inflammatory drug use** | Yes | 20 (8.8) | 12 (2.6) | 3.67 (1.76-7.65) | 0.001 |
|  | No | 208 (91.2) | 458 (97.4) | 1 |  |
| **Early childhood severe respiratory infection** | Yes | 35(15.4) | 41(8.7) | 1.89( 1.17-3.07) | 0.009 |
|  | No | 193(84.6) | 429(91.3) | 1 |  |
