# Supplementary material for: Determinants of asthma among adults in Tigray, Northern Ethiopia: a facility-based case-control study
Source: PeerJ. 2024 Jan 5;12:e16530. doi: 10.7717/peerj.16530 (PMC10773448; doi:10.7717/peerj.16530)
Supplement: Table S3 [file peerj-12-16530-s003.docx]

**Table 3: Bivariate Logistic regression analysis of Environmental and Behavioral variables assessed as determinants of Asthma among adults in Tigray Hospitals, Northern Ethiopia, 2019 (n=698)**

| **Variables** | **Category** | **Asthma Status** | | **COR [95 % CI]** | **P value** |
| --- | --- | --- | --- | --- | --- |
|  |  | **Cases (%)** | **Controls n (%)** |  |  |
| **Exposed to Dust or smoke**  **in the house** | Yes | 95 (54) | 81 (46) | 3.43 (2.40-4.89) | 0.000 |
|  | No | 133 (25.5) | 389 (74.5) | 1 |  |
| **Exposed to Dust or smoke outside the house** | Yes | 60 (26.3) | 76 (16.2) | 1.85 (1.26-2.72) | 0.002 |
|  | No | 168(73.7) | 394 (83.8) | 1 |  |
| **Firewood/coal use** | Yes | 177 (77.6) | 270 (57.4) | 2.57 (1.79-3.69) | 0.000 |
|  | No | 51(22.4) | 200 (42.6) | 1 |  |
| **Road traffic proximity from your house in minute** | <2 Minutes | 62 (27.2) | 93 (19.8) | 1.51 (1.05-2.19) | 0.022 |
|  | > 2 minutes | 166 (72.8) | 377 (80.2) | 1 |  |
| **Door open while cooking** | Yes | 92 (40.4) | 104(22.1) | 1 |  |
|  | No | 136 (59.6) | 366 (77.9) | 0.42(0.29-0.59) | 0.000 |
| **Any Dampness in the house** | Yes | 34(14.9) | 38(8.1) | 1.99 (1.22-3.26) | 0.006 |
|  | No | 194(85.1) | 432(91.9) | 1 |  |
| **Having a separate room for cooking** | Yes | 80 (35.1) | 130 (27.7) | 0.71 (0.50-0.99) | 0.024 |
|  | No | 148 (64.9) | 340(72.3) | 1 |  |
| **Possession of pets** | Yes | 56 (24.6) | 23 (4.9) | 6.33(3.78-10.60) | 0.000 |
|  | No | 172 (75.4) | 447 (95.1) | 1 |  |
| **Physical Inactivity** | Yes | 57(25) | 75(16) | 1.76 (1.19-2.59) | 0.005 |
|  | No | 171(75) | 395(84) | 1 |  |
| **Packed food use** | Yes | 43(18.9) | 56(11.7) | 1.72 (1.11-2.65) | 0.014 |
|  | No | 185(81.1) | 414(88.1) | 1 |  |
